# Electrocardiographic Changes Associated with Smoking and Smoking Cessation: Outcomes from a Randomized Controlled Trial

**DOI:** 10.1371/journal.pone.0062311

**Published:** 2013-04-23

**Authors:** Adam D. Gepner, Megan E. Piper, Miguel A. Leal, Asha Asthana, Michael C. Fiore, Timothy B. Baker, James H. Stein

**Affiliations:** 1 Division of Cardiovascular Medicine, Department of Medicine, University of Wisconsin School of Medicine and Public Health, Madison, Wisconsin, United States of America; 2 Department of Medicine, Center for Tobacco Research and Intervention, University of Wisconsin School of Medicine and Public Health, Madison, Wisconsin, United States of America; University Hospital Freiburg, Germany

## Abstract

**Introduction:**

Cardiovascular disease (CVD) can be detected and quantified by analysis of the electrocardiogram (ECG); however the effects of smoking and smoking cessation on the ECG have not been characterized.

**Methods:**

Standard 12-lead ECGs were performed at baseline and 3 years after subjects enrolled in a prospective, randomized, placebo-controlled clinical trial of smoking cessation pharmacotherapies. ECGs were interpreted using the Minnesota Code ECG Classification. The effects of (i) smoking burden on the prevalence of ECG findings at baseline, and (ii) smoking and smoking cessation on ECG changes after 3 years were investigated by multivariable and multinomial regression analyses.

**Results:**

At baseline, 532 smokers were (mean [SD]) 43.3 (11.5) years old, smoked 20.6 (7.9) cigarettes/day, with a smoking burden of 26.7 (18.6) pack-years. Major and minor ECG criteria were identified in 87 (16.4%) and 131 (24.6%) of subjects, respectively. After adjusting for demographic data and known CVD risk factors, higher pack-years was associated with major ECG abnormalities (p = 0.02), but current cigarettes/day (p = 0.23) was not. After 3 years, 42.9% of subjects were abstinent from smoking. New major and minor ECG criteria were observed in 7.2% and 15.6% of subjects respectively, but in similar numbers of abstinent subjects and continuing smokers (p>0.2 for both). Continuing smokers showed significant reduction in current smoking (–8.4 [8.8] cigarettes/day, p<0.001) compared to baseline.

**Conclusions:**

In conclusion, major ECG abnormalities are independently associated with lifetime smoking burden. After 3 years, smoking cessation was not associated with a decrease in ECG abnormalities, although cigarettes smoked/day decreased among continuing smokers.

## Introduction

Cigarette smoking contributes to the loss of over 5 million life-years annually in the United States [1]. Nearly 20% of all coronary heart disease deaths can be attributed to smoking [1], [2]. Smoking, therefore, is one of the most important modifiable risk factors for cardiovascular disease (CVD) and myocardial infarction [3].

Cigarette smoking leads to hypoxemia and endothelial dysfunction which accelerate atherosclerotic changes, increasing smokers’ risk for CVD [4], [5]. The 12-lead electrocardiogram (ECG) is a routine, inexpensive tool for assessment of CVD and CVD risk in both clinical and research settings, and ECG changes powerfully predict future CVD events [6], [7]. Large, population-based studies have described a higher prevalence of smoking in subjects with ECG abnormalities and demonstrated that ECG abnormalities are associated with increased CVD events and death [8], [9]. Although the strong relation between smoking and CVD is well-established [10], [11], there are relatively few data that prospectively described ECG changes following a smoking cessation attempt. Furthermore, most of the existing data about CVD and smoking is from older cohorts that are not representative of today’s smokers, who tend to be more overweight, are more likely to be female, and to have lower socioeconomic status [12]. To our knowledge, no studies have described the prevalence of ECG abnormalities in a younger group of cigarette smokers or their associations with smoking burden. The primary aim of this study was to describe the prevalence and magnitude of resting ECG abnormalities among smokers and to explore the relation between smoking burden and ECG abnormalities. A secondary aim was to determine if successful smoking cessation reduced, or if continued smoking increased the incidence of ECG abnormalities that indicate structural or conduction system function, after three years.

## Methods

### Ethics Statement

The institutional review board at the University of Wisconsin School of Medicine and Public Health approved this study. It was conducted according to the principles expressed in the Declaration of Helsinki. All subjects provided written informed consent.

### Study Participants and Design

All subjects agreed to participate in a randomized, double blind, placebo-controlled trial to evaluate the efficacy of smoking cessation pharmacotherapies and natural history of continued smoking and smoking cessation (clinicaltrials.gov registration no. NCT00332644) [13]. Specific recruitment strategies have been described previously [13]. This article describes a pre-specified secondary analysis of the data from the baseline and year 3 (final) study visits regarding the effects of smoking and smoking cessation on ECG changes at baseline and 3 years after the target quit date.

Major inclusion criteria were: age ≥18 years, smoking ≥10 cigarettes/day (cpd), expired carbon monoxide (CO) level >9 ppm, and stated motivation to quit smoking. Exclusion criteria have been reported previously [13]; the major reasons for exclusion were blood pressure (BP) >160/100 mmHg, myocardial infarction within the previous 4 weeks, heavy alcohol use, history of seizure or serious head injury, use of contraindicated medications, and current pregnancy or breast-feeding [13].

### Study Procedures

Subjects were recruited from communities in and around Madison and Milwaukee, Wisconsin from January, 2005 to June, 2007 [13]. Only subjects recruited from Madison, Wisconsin had resting ECGs obtained prior to an exercise stress test that was used to evaluate functional capacity [14]. Of those participants, 541 had a resting ECG available for coding; 3 did not include a subject ID and 6 could not be coded due to tracing artifact. Similarly, of the 370 subjects at 3 years of follow up, 355 ECGs were available for coding and 1 could not be coded due to tracing artifact. The baseline clinical trial visits included measurement of anthropometric data, fasting laboratory tests, completion of validated questionnaires, interviews, and resting 12-lead ECGs. Smoking burden was evaluated by current cigarette smoking in cigarettes per day (cpd) and pack-years (current cpd * years smoked). Recent smoke exposure was measured by an exhaled CO level, which reflects smoking efficiency and recent smoke exposure. Smoking status (abstinence or continued smoking) was assessed by self-reported 7-day point-prevalence abstinence using a smoking calendar and the timeline follow-back method, and then confirmed by exhaled CO levels of <10 ppm (Micro-3 Smokerlyzer, Bedfont Scientific, Williamsburg, VA) [13]. Fasting blood samples were obtained by venipuncture and refrigerated.

### Electrocardiographic Data

Participants had a 12-lead ECG recorded in the resting supine position using a standardized acquisition procedure at baseline and 3 years after a target quit day. Electrocardiograms were reviewed by 2 independent readers (ADG, MAL) and coded according to the Minnesota Code (MC) criteria [15]. Any discordant results were reviewed and adjudicated by a board certified cardiologist (JHS). All ECG reviewers were blinded to smoking status. The QRS axis and intervals (RR, PR, QRS, QT, and corrected QT) were measured manually and recorded. ECG abnormalities were categorized as major or minor, using similar criteria as used by the pooling project [16]. Major ECG abnormalities included major Q waves (MC 1-1, 1-2), significant ST segment depression (MC 4-1, 4-2), deep T wave inversion (MC 5-1, 5-2), complete or type II second-degree atrioventricular block (MC 6-1, 6-2), complete left or right bundle branch block, or intraventricular block (MC 7-1,7-2,7-4), atrial fibrillation/flutter (8-3), left ventricular hypertrophy (3-1) and right or left atrial enlargement (9-3, 9-6). Minor ECG abnormalities included borderline Q waves (MC 1-3), borderline ST segment depression (MC 4-3), moderate T wave inversion (MC 5-3), first-degree AV block (MC 6-3), type I second degree heart block (MC 6-2-3), incomplete bundle branch block (MC 7-3, 7-6), frequent premature beats (MC 8-1, 8-2), high voltage QRS (MC 3-1, 3-2), and axis deviation (MC 2-1, 2-2). ECGs without major or minor abnormalities were considered normal. ECG abnormalities for each subject were tallied and reported at baseline and at year 3. New ECG abnormalities were defined as ECG abnormalities at year 3 that were not present at baseline. Suppression codes, as detailed by the MC criteria, were used to eliminate incompatible codes within each ECG[15].

### Statistical Analysis

All analyses were conducted using SPSS Statistics 20.0 software (SPSS Inc, Chicago, Illinois). Means (standard deviations) and ranges were determined for the subject characteristics and smoking intensity parameters in Table 1. Two-tailed t-tests or chi-square tests were used to compare differences in anthropomorphic data, physiological data, demographics, and smoking parameters between continuing smokers and abstainers at year 3 (Table 2). Multivariable logistic regression models were constructed to determine associations between ECG abnormalities, at baseline and at 3 years after the target quit date, with measures of smoking burden. These models controlled for baseline age, sex, race, weight, body-mass index, glucose, low-density lipoprotein cholesterol, educational status, use of lipid-lowering medications and use of antihypertensive medications. Multinomial regression models were used to evaluate progression and regression of ECG changes in smokers and abstainers.

**Table 1:** Subject Characteristics at Baseline and Year 3.

	Baseline N = 532		Year 3 N = 354		
	Mean(standard deviation)	Range	Mean(standard deviation)	Range	p-value
Age (years)	43.3 (11.5)	18.0–78.0	48.2 (11.3)	21.9–77.8	**<0.001**
Sex (% female)	60.3%	–	61.8%	–	0.752
Race (% white)	94.9%	–	98.5%	–	**0.004**
Body-mass index (kg/m2)	28.4 (6.0)	15.7–53.1	29.8 (6.9)	16.3–60.2	**<0.001**
Waist circumference (cm)	94.9 (14.9)	63.0–142.0	97.8 (16.5)	66.0–150.0	**<0.001**
Weight (kg)	82.6 (19.6)	43.7–154.3	86.0 (21.5)	45.9–175.1	**<0.001**
Systolic blood pressure (mmHg)	119.6 (13.9)	84.0–162.0	115.3 (15.7)	78.0–178.0	**<0.001**
Diastolic blood pressure (mmHg)	75.8 (8.9)	48.0–100.0	68.8 (10.3)	42.0–108.0	**<0.001**
Use of antihypertensive medications (%)	25 (4.7%)	–	53 (17.1%)	–	**<0.001**
Smoking burden (current pack-years)	26.7 (18.6)	1.0–112.5	27.6 (17.8)	1.1–117.0	**<0.001**
Alcohol quantity frequency measure	19.5 (27.6)	19.5–168.0	19.8 (32.8)	0.0–280.0	0.388
Fasting Glucose (mg/dL)	94.0 (13.0)	57.0–201.0	99.5 (18.9)	70.0–263.0	**<0.001**
Hemoglobin A1C(%)	5.48 (0.5)	5.0–10.0	5.74 (.53)	4.0–9.0	**<0.001**
Use of lipid-lowering medications (%)	15 (2.8%)	–	60 (19.4%)	–	**<0.001**
Total cholesterol (mg/dL)	179.0 (34.1)	76.5–288.6	186.9 (37.2)	71.5–336.3	**0.001**
Low-density lipoprotein cholesterol (mg/dL)	112.2 (28.8)	25.7–210.0	115.2 (31.1)	24.5–253.8	0.233
High-density lipoprotein cholesterol (mg/dL)	45.3 (13.7)	18.1–100.5	50.0 (14.7)	23.6–96.0	<**0.001**
Triglycerides (mg/dL)	135.9 (90.2)	33.8–887.9	140.0 (90.9)	39.8–712.9	0.431
Major ECG abnormalities	87 (16.4%)	–	51 (14.4%)	–	–
Minor ECG abnormalities	131 (24.6%)	–	82 (23.1%)	–	–
**Markers of smoking intensity**	–	–	–	–	–
Carbon monoxide (ppm)	27.2 (13.9)	2.0–110.0	11.6 (11.6)*	0.0–57.0	**<0.001**
Current smoking (cpd)	20.6 (7.9)	7.0–50.0	7.2 (9.0)**	0.0–42.9	**<0.001**

ECG = electrocardiogram cpd = cigarettes per day.

* Carbon monoxide levels for the current smokers at year 3 was 19.1(11.0) ppm.

** Cigarettes per day for the current smokers at year 3 was 13.5 (8.2) cpd.

**Table 2:** Comparison of Continuing Smokers and Abstainers at year 3.

	Year 3 N = 354		
	Continued Smokers (n = 201)	Abstainers (n = 153)	p-value
Total major ECG criteria (%)	28 (13.9%)	23 (15.0%)	0.770
Total minor ECG criteria (%)	49 (24.4%)	33 (21.6%)	0.535
New major ECG Criteria at year 3 (%)	13 (6.5%)	9 (5.9%)	0.208
New minor ECG Criteria at year 3 (%)	24 (11.9%)	16 (10.5%)	0.789
Age (years)	48.1 (10.8)	48.5 (11.8)	0.745
Sex (% female)	64.7%	57.6%	0.186
Race (% white)	94.7%	97.2%	0.603
Body-mass index (kg/m2)	29.0 (6.6)	30.9 (7.2)	**0.008**
Waist circumference (cm)	95.7 (16.0)	100.5 (16.7)	**0.006**
Weight (kg)	82.6 (20.1)	90.4 (22.5)	**0.001**
Systolic blood pressure (mmHg)	114.2 (16.6)	116.6 (14.5)	0.175
Diastolic blood pressure (mmHg)	67.9 (10.5)	69.9 (10.0)	0.080
Use of antihypertensive medications (%)	15.3	19.5	0.320
**Markers of smoking intensity**	–	–	–
Carbon monoxide level (ppm)	18.9 (10.8)	2.2 (1.3)	**<0.001**
Current smoking (cpd)	12.4 (8.8)	0.3 (1.5)	**<0.001**
Smoking burden (current pack-years)	28.9 (18.0)	26.0 (17.5)	0.150
Alcohol quantity frequency measure	17.4 (33.9)	22.9 (31.1)	0.164
Fasting Glucose (mg/dL)	98.7 (18.3)	100.6 (19.6)	0.350
Hemoglobin A1C (%)	5.7 (0.5)	5.7 (0.6)	0.714
Use of lipid-lowering medications (%)	18.6	20.3	0.715
Low-density lipoprotein cholesterol (mg/dL)	113.9 (30.2)	116.8 (32.2)	0.401
High-density lipoprotein cholesterol (mg/dL)	49.4 (14.4)	50.9 (15.1)	0.340
Triglycerides (mg/dL)	139.9 (100.1)	140.0 (77.3)	0.995

ECG = electrocardiogram cpd = cigarettes per day.

## Results

### Subject Characteristics

**Figure 1:**
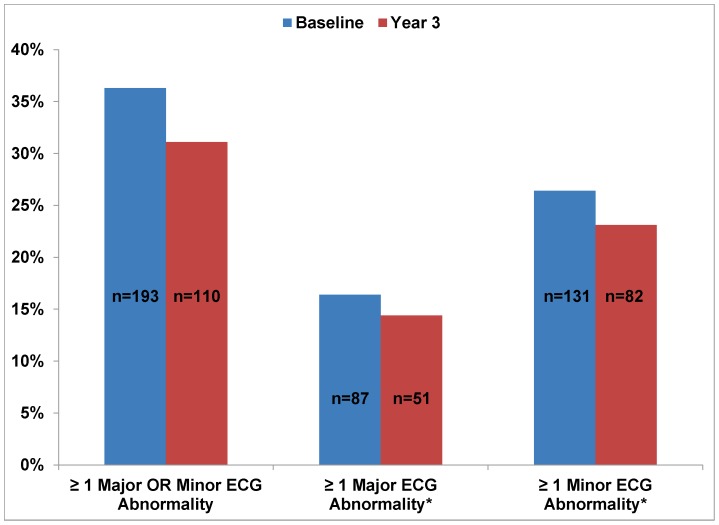
Prevalence of Major and Minor ECG Abnormalities. The percentage of subjects with major and minor ECG abnormalities at baseline (blue) and at year 3 (red). Year 3 percentages include both continued smokers and those who abstained. All p-values for comparisons between baseline and year 3 ECG abnormalities were non-significant (<0.05). ECG = electrocardiogram. *Subjects may have had more than one major and/or minor ECG abnormalities. Each abnormality was tallied separately.

**Figure 2:**
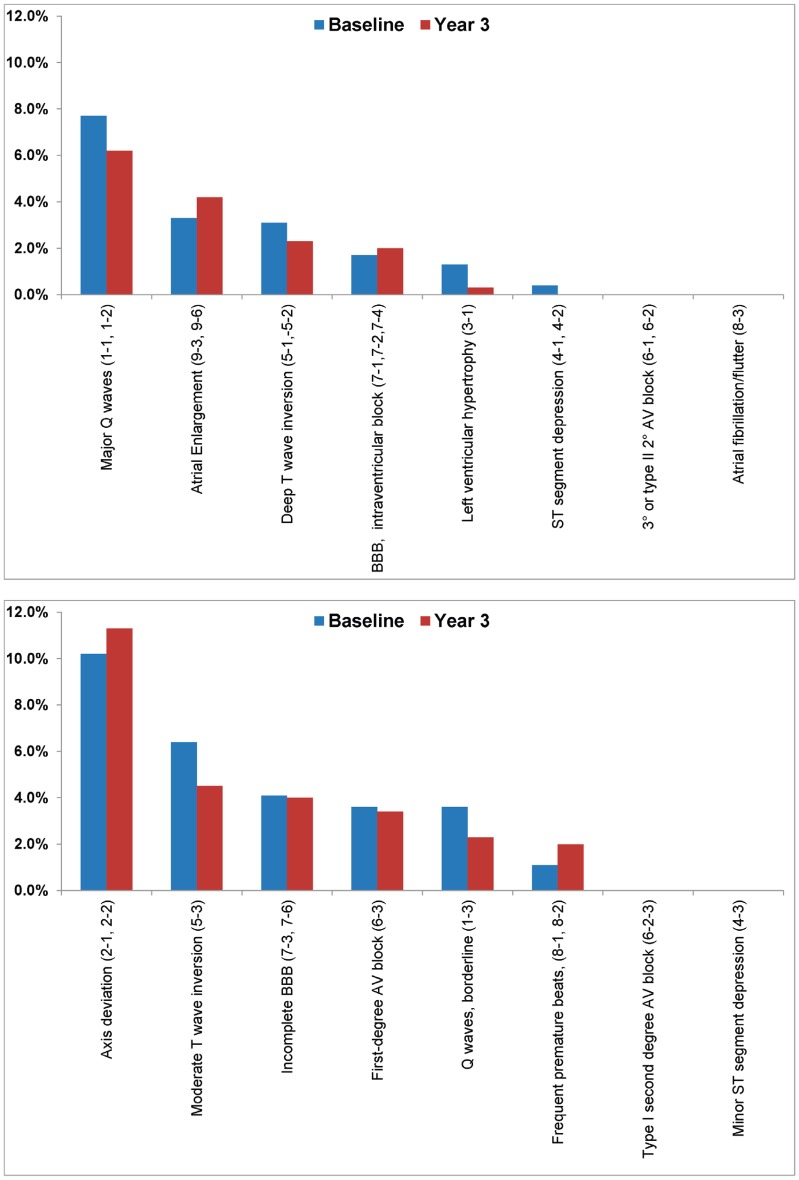
(A). Prevalence of Specific Major ECG Abnormalities. (B). Prevalence of Specific Minor ECG Abnormalities. Figure 2A shows percentages of the specific major ECG abnormalities and figure 2B shows percentages of the specific minor ECG abnormalities at baseline (blue) and at year 3 (red). Year 3 percentages include both continued smokers and those who abstained. All p-values for comparisons between baseline and year 3 ECG abnormalities were non-significant (<0.05). BBB = Bundle Branch Block; AV = Atrioventricular.

Subject characteristics at baseline and year 3 are provided in Table 1. At baseline, resting ECGs were obtained in 532 current smokers. Of these subjects, 63% were women, 95% were white and 3.2% were African-American. They were (mean [SD]) 43.3 (11.5) years old and smoked 20.6 (7.9) cpd with a smoking burden of 26.7 (18.6) pack-years. Blood pressure (119.6 [13.9]/75.8[8.9] mmHg) and fasting lipid values were normal. At baseline, only 25 (4.7%) of the subjects were on antihypertensive therapy and only 15 (2.8%) of the subjects were using lipid-lowering medications (Table 1). Major and Minor ECG abnormalities at baseline and year 3 are shown in Figures 1 and 2. At baseline, 87 subjects (16.4%) had at least one major ECG abnormality and 131 (24.6%) had at least one minor abnormality. The most prevalent major ECG abnormalities at baseline were major Q waves (MC 1-1, 1-2), observed in 41 (7.7%) subjects, deep T-wave inversion (MC 5-1, 5-2) in 17 (3.1%) subjects, and atrial enlargement (MC 9-3, 9-6) in 18 (3.3%) subjects. The most frequent minor ECG abnormalities at baseline were axis deviation (MC 2-1, 2-2) in 58 (10.9%) subjects, moderate T-wave inversion (MC 5-3) in 34 (6.4%) subjects, incomplete bundle branch block (MC 7-3, 7-6) in 22 (4.1%) subjects, 1st degree atrioventricular block (MC 6-3) in 19 (3.6%) subjects and borderline Q waves (MC 1-3) in 18 (3.6%) of subjects.

### ECG Abnormalities and their Relationship with Smoking Parameters

Greater pack-years was strongly associated with major ECG abnormalities (p = 0.001; OR = 1.21, 95% CI = 1.07–1.36 per 10 pack-years), however cpd (p = 0.150; OR = 1.22; 95% CI = 0.92–1.61 per 10 cpd) and exhaled CO (p = 0.379; OR = 1.07, 95% CI = 0.92–1.25 per 10 ppm) were not. After adjusting for age, sex, race, education, body-mass index, fasting glucose, and lipids, greater pack-years remained associated with major ECG criteria (p = 0.016; OR = 1.23, 95% CI = 1.03–1.47 per 10 pack-years). After adjusting for confounders, there were no significant associations with minor ECG abnormalities and smoking burden with respect to cpd (p = 0.46; OR = 1.11, 95% CI = 0.84–1.45 per 10 cpd), exhaled CO (p = 0.52; OR = 1.05; 95% CI = 0.92–1.21 per 10 ppm) or pack-years (p = 0.70; OR = 1.03; 95% CI = 0.88–1.20 per 10 pack-years). Results were similar for the unadjusted analyses. To ensure that the association between pack-years and major ECG changes was not simply due to atrial enlargement (MC 9-3, 9-6) since this was included as a major criteria in the pre-specified analysis plan but not the pooling project [16], the analyses were performed including atrial enlargement as minor ECG criteria. Still, major ECG abnormalities were significantly associated with pack-years (p = 0.001; OR = 1.21, 95% CI = 1.08–1.36 per 10 pack-years) and minor criteria were not (p = 0.14; OR = 1.08, 95% CI = 0.98–1.19 per 10 pack-years).

### Longitudinal Changes in ECG Abnormalities after a Quit Attempt

Of the 354 subjects with ECGs at year 3, abstinence from smoking was confirmed in 153 (43%) participants by CO level <10 ppm. As is often observed in longitudinal smoking cessations studies [17], [18], there was moderate attrition after 3 years with 119 (34%) subjects not returning for that visit. Several important differences between the baseline and year 3 data were observed amongst those completing follow-up. Subjects who followed up at year 3 were more likely to be white (p = 0.004) and on average, were 3.6 kg heavier (p<0.001) and had 2.9 cm larger waist circumferences (p<0.001). Additionally, those who followed up had fasting glucose and hemoglobin A1C levels that were 4.5 mg/dL and 0.26% higher respectively (both p<0.001). Systolic and diastolic blood pressures were 4.3 and 7.0 mmHg lower in those who followed up (both p<0.001), and a higher percentage were being treated with antihypertensive medications (p<0.001). A higher percentage of subjects who were followed up at year 3 were on lipid-lowering therapy (p<0.001), although there was not a significant difference in LDL-cholesterol levels (Table 1).

Major Q waves (MC 1-1, 1-2) also were the most frequent major ECG abnormality at year 3 (22 [6.2%] subjects), followed by atrial enlargement (MC 9-3, 9-6; 15 [4.2%] subjects) and deep T wave inversion (MC 5-1, 5-2; 8 [2.3%] subjects) (Figure 2A). Axis deviation (MC 2-1, 2-2; 40 [11.3%] subjects) and moderate t-wave inversion (MC 5-3; 16 [4.5%] subjects) remained the most common minor ECG changes at year 3 (Figure 2B). New major and minor ECG criteria were observed in 7.2% and 15.6% of subjects respectively, but in a similar number of abstinent subjects and continuing smokers (p>0.2 for both, Table 2). Abstinent subjects gained significantly more weight than subjects who continued to smoke over the 3 year follow up period (5.95 [9.2] kg vs. 1.74 [7.8] kg, p<0.001). Compared to baseline, continuing smokers at year 3 smoked significantly fewer cigarettes (-8.4 [8.8] cpd) (p<0.001). After adjusting for baseline pack-years, multinomial regression models revealed no differences in progression (p = 0.727; OR = 1.17, 95% CI = 0.48–2.82) or regression (p = 0.081; OR = 2.26, 95% CI = 0.91–5.47) of major ECG abnormalities in smokers and abstainers. Similarly, for minor ECG abnormalities, there were no differences in progression (p = 0.665; OR = 1.16, 95% CI = 0.59–2.31 or regression (p = 0.610; OR = 1.20, 95% CI = 0.60–2.41) between smokers and abstainers at year 3. Additionally, no sex or racial differences were observed.

## Discussion

In the general population, major and minor ECG changes predict increased mortality [7], [19]. Individuals who smoke are more likely to have ECG findings consistent with ischemic heart disease [20], structural heart disease [21], and cardiac rhythm disorders [22]. Despite, on average, being nearly a decade younger than participants in population-based studies, the smokers in our study had notably more major ECG abnormalities (especially major Q waves) than has been observed in the general population [8], [23]–[25]. Participants in the Coronary Artery Risk Development in Young Adults (CARDIA) study, a large epidemiological study that reported a cross-sectional evaluation of ECG abnormalities in subjects of a comparable age to those in our cohort, had 60% fewer major Q wave findings (MC 1-1 and 1-2) [26]. Only 14% of comparable CARDIA subjects were current smokers, indicating that smokers have a higher prevalence of major Q wave findings [26]. Indeed, in CARDIA, active smokers had a higher prevalence of major and minor Q-waves than non-smokers, though no information regarding the smoking burden of CARDIA subjects was reported [26]. In larger epidemiological studies that evaluated ECG abnormalities, the majority of subjects studied were one to two decades older that those in our study. In those studies, a minority of subjects were active smokers [7], [23], [25], [27], [28] or smokers were excluded from the analyses [29]. The ECG abnormalities in younger smokers have not been well-described and are in need of further study. Considering that age is one of the strongest predictors of 10-year CVD risk [30] the age-independent association between baseline major Q-waves and smoking burden in pack-years supports the idea that increased lifetime smoking burden increases CVD risk and that increased CVD risk related to long-term smoking can be detected by the 12-lead ECG. There was no independent relationship between ECG abnormalities and cpd at baseline, however sudden cardiac death in smokers also has been associated with the overall duration of smoking, but not cpd [31].

While there were fewer minor ECG abnormalities in those who quit smoking, we did not observe a significant difference in major or minor ECG findings at year 3. This is not unexpected since Q-waves, our most common major finding, are indicative of myocardial scar which is unlikely to resolve during our 3 year follow up period [32]. It is possible that the length of follow up was inadequate to detect differences in ECG progression or regression. Still, prior studies have shown that with improved blood pressure control, ECG abnormalities can regress within 2–5 years [32], [33]. Longitudinal data regarding ECG changes after a quit attempt have not been described previously. Also, continuing smokers in our study were smoking significantly fewer cigarettes than they were at baseline and this group gained less weight then abstainers, which would be expected to attenuate the effects of smoking on ECG abnormalities. Additionally, there was a significant increase in use of antihypertensive medications and lipid-lowering therapy at year 3, compared to baseline. Treatment of known CVD risk factors could decrease the likelihood of developing ECG abnormalities at year 3 regardless of smoking status. For example, the prevalence of ECG abnormalities suggestive of left ventricular strain, such as T-wave inversion and LVH, slightly decreased at year 3 in both groups. This is more likely due to improvements in blood pressure rather than changes in smoking [32], [33].

### Limitations

Because this was a randomized clinical trial of smoking cessation interventions, there are no nonsmoking controls for comparison. In smoking cessation studies, it is very common for subjects who relapse to drop out or miss follow-up visits [17], [18]. In our study, 34% of subjects did not have a 3-year ECG, which is consistent with the 30–43% one year drop-out rates reported in recent clinical trials of smoking cessation pharmacotherapies [17], [18]. There were differences in participants that did not return for follow up with regard to age, and race which may introduce selection bias. Though, those who did not attend follow-up smoked a similar number of cigarettes/day at baseline [5], [12]. The three year follow up period in this study may be too short to detect progression or regression of some ECG changes [32], [33]. Finally, although ECG changes are associated with increased mortality, hard CVD outcomes including death, were not assessed in this study population.

### Conclusions

Even in relatively young adults, major ECG abnormalities are independently associated with smoking burden. Minor ECG abnormalities were not associated with current or past smoking burden. After 3 years, quitting smoking was not associated with a decrease in ECG abnormalities; however those who abstained gained significantly more weight and continuing smokers smoked fewer cpd than at the inception of the study. Longer follow-up likely is needed to identify the effects of quitting versus continued smoking on the ECG and CVD risk.
